# Sugar Transport, Metabolism and Signaling in Fruit Development of *Litchi chinensis* Sonn: A Review

**DOI:** 10.3390/ijms222011231

**Published:** 2021-10-18

**Authors:** Shuying Fan, Dan Wang, Hanhan Xie, Huicong Wang, Yonghua Qin, Guibing Hu, Jietang Zhao

**Affiliations:** State Key Laboratory for Conservation and Utilization of Subtropical Agro-Bioresources/Key Laboratory of Biology and Genetic Improvement of Horticultural Crops-South China, Ministry of Agriculture and Rural Affairs, Guangdong Litchi Engineering Research Center, College of Horticulture, South China Agricultural University, Guangzhou 510642, China; fsy16616682196@126.com (S.F.); danywang1@163.com (D.W.); xie199412345@163.com (H.X.); wanghc1972@263.net (H.W.); qinyh@scau.edu.cn (Y.Q.); guibing@scau.edu.cn (G.H.)

**Keywords:** *Litchi chinensis*, sugar, transport, metabolism, abscission, seed development

## Abstract

*Litchi chinensis* Sonn. is an important evergreen fruit crop cultivated in the tropical and subtropical regions. The edible portion of litchi fruit is the aril, which contains a high concentration of sucrose, glucose, and fructose. In this study, we review various aspects of sugar transport, metabolism, and signaling during fruit development in litchi. We begin by detailing the sugar transport and accumulation during aril development, and the biosynthesis of quebrachitol as a transportable photosynthate is discussed. We then document sugar metabolism in litchi fruit. We focus on the links between sugar signaling and seed development as well as fruit abscission. Finally, we outline future directions for research on sugar metabolism and signaling to improve fruit yield and quality.

## 1. Introduction

In higher plants, the “source” refers to a photosynthesizing organ with a net export of assimilates, such as mature leaves, whereas the “sink” is an organ that consumes or accumulates assimilates, such as fruits. Photoassimilates are usually transported from sources to sinks as simple sugars, typically as sucrose. Communication between sources and sinks during plant growth and development, which determines the partitioning of carbohydrates, has a pivotal role in controlling crop yield and affecting fruit quality [1]. Sink strength is regulated by a complex signaling network encompassing sugars, hormones, and environmental factors [2]. Fruits, which are strong sinks, compete for assimilates to ensure acceptable fruit production and quality [3]. On the other hand, fruit set and development is affected by source-sink interactions. Sugars provide energy and carbon backbone, and also work as signal molecules to control fruit growth and development [4,5]. The partitioning of assimilates in fruits is regulated by several processes, including phloem transport, metabolism, and sugar signaling, by which they control sugar-mediated responses based on cellular sugar homeostasis.

Litchi (*Litchi chinensis* Sonn.) belongs to the Sapindaceae family and is an important evergreen fruit crop cultivated in the tropical and subtropical regions of the world. Litchi fruits are characterized by a succulent edible pulp (aril) with a dark brown seed at the center and a corky pericarp on the outside [6]. The outer pericarp changes to a pink/red color during fruit ripening because of anthocyanins accumulation [7]. Inside, its pulp is semi-translucent to white, which accumulates sugars amounting to 15–20% of the fresh mass [8,9]. Sucrose, fructose, and glucose are the major sugars in the pulp, and the sugar content and composition vary considerably among cultivars [10,11]. Sugar accumulation not only contributes to the sweetness and flavor of litchi fruits, but it also provides carbon and energy for fruit set, development, and ripening. This review focuses on the recent progress in the mechanism of sugar transport, phloem unloading, and metabolism. We then review how sugar signaling affects seed development and fruit abscission.

## 2. Sugar Accumulation in Litchi Fruit

### 2.1. Litchi Fruit Development

The typical litchi ovary is two-lobe shaped, with one anatropous ovule in each lobe. After fertilization, usually only one lobe develops into a single-seeded fruit, and the other one remains attached as a tiny appendix. The two ovary lobes develop into twin full size fruits in rare cases [12]. Fruit growth stages are typically sigmoidal and two distinct phases are distinguished [13]. Stage I is mainly characterized by the growth of pericarp and seed coat. Stage II is mainly characterized by the growth of an embryo (Stage II a) and the rapid aril growth and maturation (Stage II b). Stage II a is not distinct in fruit with an aborted seed. Evidence indicate that there is close correlation among the development of the pericarp, seed, and aril. Huang and Xu [14] propose the “ball-skin vs. bladder effect” concept. As the growth of the pericarp (ball skin) precedes the growth of the aril (bladder), the pericarp determines and restricts aril growth and therefore fruit size [15]. The pericarp becomes thinner, driven by the extending aril. Fruit size depends mostly on the number of pericarp cells rather than size [16].

**Figure 1:**
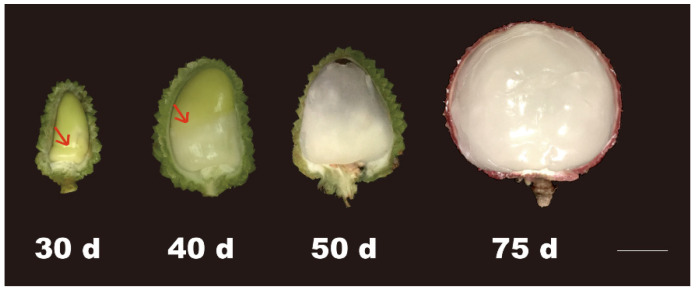
Phenotype changes of aril development at different stages. The arrowhead refers to the aril. Bar = 1 cm.

The aril develops from a site just above the obturator on the funicle [17]. The aril appears around 21–35 days after anthesis (DAA) as a ring of white tissue around the seed base [16]. It grows upwards, eventually enclosing the seed (Figure 1). The growth rate of aril is slow at the beginning, but increases rapidly with fruit ripening. The mature aril consists of large, irregular, thin-walled cells and contains around 15–20% dry mass.

### 2.2. Sugar Accumulation during Aril Development

Litchi fruit is widely eaten by consumers due to its sweet taste and rich nutrition [18]. Sugars are the major nutrient in litchi aril, which determine the fruit quality and flavor. Litchi fruit accumulates different types of soluble sugars and methylated cyclitols, mainly sucrose, glucose, fructose and quebrachitol (2-O-methyl-chiro-inositol) [19]. The sugar composition in the mature aril varies considerably among different litchi cultivars. According to the hexose/sucrose ratio, litchi cultivars are classified into three types: hexose prevalent type (hexose/sucrose ratios > 2), sucrose prevalent type (hexose/sucrose ratios < 1), and intermediate types (1 < hexose/sucrose ratios < 2) [9,10]. The difference in hexose/sucrose ratio between litchi cultivar offers a valuable system for the regulatory mechanisms of sugar accumulation in fruits. High levels of quebrachitol, a major sugar derivative, have been detected in litchi aril [20]. In addition to oligosaccharides, mature litchi aril also contains various polysaccharides [18].

The content of total sugar increases during aril development and maturation, decreasing during postharvest storage [21]. However, the patterns of sugar accumulation in the aril are significantly different between the hexose-prevalent type (‘Feizixiao’, FZX) and the sucrose-prevalent type (‘Wuheli’, WHL). In FZX, sucrose initially increases and then declines at 60 DAA when the fruit reaches half its mature weight, while glucose and fructose increase steadily, resulting in a high hexose/sucrose ratio. In WHL, sucrose increases during aril development up to maturity, but glucose and fructose remain constant during fruit ripening, leading to a low hexose/sucrose ratio. Quebrachitol decreases and remains constant during fruit ripening [22].

### 2.3. Sugar Transport and Sugar Transporters of Litchi Fruit

**Figure 2:**
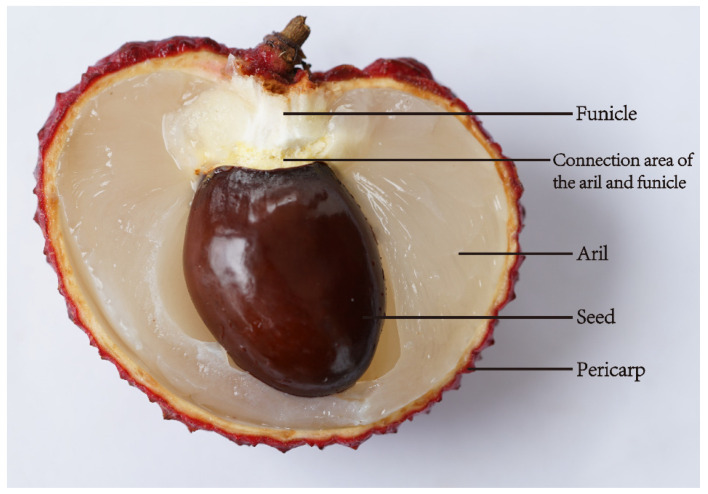
Structure of litchi fruit.

It is well recognized that phloem unloading in sink organs plays a critical role in regulating the distribution of photoassimilate [23]. Post-phloem transport has symplastic and/or apoplastic pathways depending on the type of organ and developmental state [24]. The vascular bundles of litchi fruit end in the funicle or seed stalk. There is no vascular bundle in the aril and the connection area of the aril and funicle. Therefore, the funicle, where the aril originates, serves as the connection between the vascular pedicel and the aril (Figure 2). This special structure suggests that phloem unloading into aril seems to be apoplastic. Carboxyfluorescein (CF), a cell-to-cell migration of symplastically restricted fluorescent dye, was used to test the continuity of the symplasmic pathway between the funicle and aril. CF was transported along the vascular bundle and diffused to funicle parenchyma cells, but it was confined there with no distribution in the aril [25]. Therefore, the aril is symplasmically separated from the funicle. The post-phloem sugar transportation pathway from the funicle to the aril is apoplasmic.

Sugars accumulate in the vacuoles of flesh cells by either simple diffusion along a steep concentration gradient or energy-driven transporter-mediated process. Since the post-phloem sugar transport into aril through the apoplasmic pathway, energy-driven transporters rather than the sugar gradient might play key roles in regulating aril sugar accumulation in litchi. Infiltration of both an ATPase inhibitor [eosin B (EB)] and a sucrose transporter inhibitor [p-chloromercuribenzene sulfonate (PCMBS)] inhibited sugar accumulation in litchi aril [25]. In summary, the uploading of sugars into litchi aril depends on ATPase and involves the action of the sugar transporter.

Analysis of the litchi genome sequence shows that sucrose transporters (SUTs) and hexose transporters (HTs) are both encoded by a multi-gene family. Five and seven ortholog genes of SUTs and HTs are identified in litchi. There is considerable variation in the expression patterns of *LcSUT* and *LcHT* genes in the aril. *LcSUT1*, *LcSUT4*, *LcHT2* and *LcHT5* display higher expression levels in the aril [25]. LcSUT1 shows high similarity to the SUC3/SUT2 group, while LcSUT4 shows high similarity to the SUC4/SUT4 group. During aril development, stronger expression of *LcSUT4* coincide with the sugar accumulation, suggesting that LcSUT4 is involved in apoplasmic transport for sugar accumulation in litchi aril [25]. However, the expression of *LcHT2* and *LcHT5* is not paralleled by the increase in hexose concentration in litchi aril. Further work is needed to clarify their roles in sugar accumulation in litchi fruit.

More recently, a new class of sugar transporters named Sugar Will Eventually be Exported Transporters (SWEETs) has been identified from archaebacteria to plants and humans. SWEETs can mediate both cellular uptake and efflux of various mono- and disaccharides, which is largely pH independent [26]. Sixteen *LcSWEET* genes are identified in the litchi genome, which are divided into four clades by phylogenetic tree analysis [27]. Among them, *LcSWEET10* is strongly expressed with the sugar accumulation and has different expression patterns during aril development between cultivars with different hexose/sucrose ratios (unpublished data). The similar gene in grapevine *VvSWEET10* has been proved to be a hexose-affinity transporter with broad spectrum of sugar transport functions [28].

### 2.4. Biosynthesis of Quebrachitol, a Transportable Photosynthate

In litchi, as in many higher plants, sucrose is the major photosynthetic product of long-distance transport in the phloem. Recently, quebrachitol, the main element in the aril of litchi, was identified as one of the transportable photosynthates [20]. Quebrachitol was first discovered as a natural product in *Aspidosperma quebracho* (Apocynaceae), and was later detected in various species of the Sapindaceae. Quebrachitol is ubiquitous in litchi organs and tissues, with especially high levels in leaves, phloem, and xylem. Wu et al. [20] fed ^14^CO_2_ to litchi leaves, and detected radioactivity in both the sucrose and quebrachitol fractions, proving quebrachitol is a translocated photoassimilate. The accumulation of quebrachitol might be a taxonomic trait of plants belonging to the Sapindaceae, such as sorbitol in species of the Rosaceae and mannitol in members in the Apiaceae. The high concentrations of quebrachitol in litchi may represent an important carbon metabolic strategy that maintains osmolality under reduced-sucrose conditions.

Myo-inositol is the precursor for quebrachitol synthesis, which involves the methylation of myo-inositol and subsequent epimerization of bornesitol (the methylated intermediate). In litchi, inositol 1-*O*-methyltransferase has been identified as a catalyzer the methylation of myo-inositol to the formation of bornesitol (1-*O*-methyl-myo-inositol). Wu et al. [20] identified an inositol methyltransferase gene (*LcIMT1*) involved in bornesitol biosynthesis. The down-regulation of *LcIMT1* through virus-induced gene silencing (VIGS) resulted in significantly lower concentrations of bornesitol. These results suggest that bornesitol is a stable intermediate in quebrachitol biosynthesis.

## 3. Sugar Metabolism in Litchi Fruit

Once imported into sink cells, sucrose is either stored or metabolized. Sucrose synthases (SuSy: EC 2.4.1.13) and invertases (INV: EC 3.2.1.26) are mainly involved in the cleavage of sucrose, which helps the maintenance of sink strength [29]. In addition, re-synthesis of sucrose in sink cells may occur via sucrose phosphate synthase (SPS: 2.4.1.14).

### 3.1. SPS

SPS is a key enzyme of sucrose synthesis from Fructose-6-Phosphate and uridine diphosphate (UDP)-glucose. Its activity has been shown to be associated with plant biomass production. In sugarcane, overexpression of *SoSPS1* gene increased plant biomass production and stalk [30]. In litchi, no significant correlation was observed between the aril hexose/sucrose ratio and SPS activity [10]. However, the activities of SPS and expression levels of *LcSPS* genes in high-sucrose cultivar WHL were much higher than those in low-sucrose cultivar FZX [9]. Indeed, sugar accumulation and composition in fruit comprises a complex regulatory network [31]. Sucrose can be resynthesized in sink cells, particularly where phloem unloading occurs apoplasmically [32]. Higher activities and expression levels of *LcSPSs* in WHL indicate that SPS might be involved in the re-synthesis of sucrose to maintain high sucrose accumulation in aril with low hexose/sucrose ratios.

### 3.2. SuSy

SuSy catalyzes the reversible cleavage of sucrose and UDP into fructose and UDP-glucose. It has been proposed to be a biochemical marker of sink strength. In addition, increasing evidence suggest that SuSy functions in sink development and responds to environmental changes in plants [32]. In litchi, SuSy activities increase with increasing hexose/sucrose ratios among different cultivars. There is a significantly positive linear correlation between SuSy activities and the hexose/sucrose ratio [10]. Five SuSy genes are identified in litchi, which exhibit distinct but partially redundant expression patterns [22]. The expression profile of *LcSuSy1* is consistent with the trend of sugar accumulation during aril development, suggesting it may play an important role in the determination of sink strength in aril. Moreover, *LcSuSy2*, *LcSuSy4*, and *LcSuSy5* show varied expression patterns between cultivars with different hexose/sucrose ratios [22]. These analyses highlight the diversity and complexity of SuSy function in aril development.

### 3.3. INV

INV irreversibly hydrolyzes sucrose into glucose and fructose. According to their subcellular localization, INVs are classified into three groups: cell wall invertases (CWIN), vacuolar invertases (VIN), and cytoplasmic invertases (CIN). CWIN and VIN, which are also called acid invertase, have an acidic pH optimum (4.5–5.5). By contrast, CIN has a neutral/alkaline pH optimum (7.0–7.8) [33]. INVs have a crucial function in plant growth and development, and CWIN and VIN are also involved in abiotic and biotic stress responses [5]. For example, a major CWIN gene, *LIN5*, and CWIN activity are differentially activated in styles and ovaries during pollination and fertilization, and elevated CWIN activity up-regulates sugar transporters to promote sugar uptake by parenchyma cell for early fruit development in tomatoes [34,35]. In addition, INV activity is post-translationally regulated by invertase inhibitors (INHs) in plant [36].

In litchi, the activities of CWIN and VIN in the arils are significantly and positively correlated with their hexose/sucrose ratios. Sucrose-prevalent type cultivars display very low or non-detectable levels of CWIN and VIN. By contrast, hexose-prevalent type cultivars show high activities of CWIN and VIN. There is no obvious correlation between CIN activities and hexose/sucrose ratio among different cultivars [10]. During aril development, CWIN and VIN activities are low at early stages and then continuously increase until maturity in hexose-prevalent cultivars, however, CWIN and VIN activities remain low in sucrose-prevalent cultivars [10]. CWIN has been shown to play an essential role in nonphotosynthetic organs where sucrose phloem unloading or subsequent post-phloem transport follow an apoplasmic pathway [37]. As previously mentioned, sugar transport from the funicle to the aril follow an apoplasmic pathway [25]. Therefore, CWIN plays a critical role in litchi aril development and fruit quality. By contrast, VIN plays a major role in cell expansion and hexose-accumulating organs [32]. In tomatoes, the lack of VIN activity in wild species, *Lycopersicon chmielewskii*, accumulates high levels of sucrose in mature fruit and transgenic repression of *SlVIN1* results in fruit accumulating sucrose instead of hexose [38]. The role of LcVIN in determining sugar composition in the litchi aril is worth further study.

## 4. Sugar Signaling and Fruit Development

In addition to the role as a nutrient, sugar can regulate plant development and gene expression by sugar signaling. Complex mechanistic approaches have been evolved in plants to sense different sugars, such as sucrose, hexoses, and trehalose [39]. Sugar signaling has been known to be involved in plant growth, development, and stress responses [40]. In addition, the interaction between sugars and plant hormones has also been described in plant development [41].

### 4.1. Sugar Regulation of Genes Involved in Fruit Abscission

Fruit abscission is a normal event during fruit development. However, massive fruit abscission is a major problem causing low and unstable yield in litchi. Around 5% or less of the initial female flowers can develop into mature fruit, depending on cultivars, weather, and the status of tree nutrients [16]. Endogenous hormones and carbohydrates have been applied to regulate fruit abscission in litchi [42]. In South China, overcast or rainy weather frequently occurs during litchi fruit development, leading to low photosynthetic activity and fruit set [16]. Artificial shading over the whole canopy or spraying photosynthetic inhibitors causes serious fruit abscission. Trunk-girlding treatment at full bloom is carried out to inhibit fruit abscission, which is probably due to more carbohydrate being available in source leaves, thereby strengthening the fruit in the competition of carbohydrate [43]. Therefore, it is proposed that the status of carbohydrate acts as an abscission signal perceived by fruit.

Recently, transcriptome analyses have been conducted to investigate the molecular events underlying fruit abscission in litchi. Carbohydrate deficiency-inducing treatments such as girdling plus defoliation result in 100% fruit drop of litchi, meanwhile, the genes involved in sugar degradation are upregulated and the sugar synthesis genes are downregulated [44]. Moreover, girdling plus defoliation treatment reduces the content of endogenous indole-3-acetic acid (IAA) and increases the transcript level of *LcAUX/IAA1*, *LcGH3.1* and *LcSAUR1*, in contrast to the decreasing level of *LcARF1* [45]. These results suggest that carbohydrate deficiency not only induce the sugar signaling, but also the endogenous hormones signals, such as auxin polar transport and signal transduction. Similarly, the expression of genes related to IAA synthesis and transport are significantly reduced in the pericarp of abscising mango fruitlets [46].

### 4.2. Sugar and Seed Development

Based on the type of seed development, litchi fruits can be categorized into three types: normal, abortive (shriveled) and seedless [16]. Seedless fruit are caused by embryo sac sterility and therefore the absence of fertilization (parthenocarpy). The aborted seeds are also called stenospermocarpy, which is caused by embryo abortion after fertilization. Embryo abortion is believed to be a consequence of both the genotype and environment, especially the temperature. There is a strong correlation between ‘Guiwei’ seed development and the minimum temperature under field conditions, indicating thermos-sensitive sterility underlying the partial seed abortion in ‘Guiwei’ [47]. Seed development depends on the coordinated development of the seed coat, endosperm, and embryo [48]. In normal-seeded cultivar, ‘Heiye’ (HY), visible liquid endosperm is observed around 21 DAA and most abundant at 28 DAA when the embryo reaches the heart stage with a rudimentary cotyledon, then the liquid endosperm is absorbed by the developing cotyledon. In the abortive-seeded cultivar, ‘Nuomici’ (NMC), visible liquid endosperm is not obvious and zygote development is retarded [49]. These results indicate that the failure of endosperm development ultimately causes the arrest of embryo development. Indeed, the endosperm acts not only as a nutrients source but also as an integrator of seed development [50]. It confirms that seed size is affected by the timing of endosperm cellularization in maize [51].

As mentioned earlier, litchi cultivars are grouped into three types according to the difference of hexose/sucrose ratio [10]. Interestingly, most of the cultivars with lower hexose/sucrose ratio have abortive seeds, and further analysis indicates that there is a significant positive correlation between hexose/sucrose ratio and seed weight [52]. Similar to the aril, the CWIN activities in the funicle and seed coat are significantly lower in NMC than in HY, which is consistent with the lower fructose and glucose contents in the funicles of NMC compared with those of HY [49]. The aril and the seed share the same phloem unloading pathway. The funicles are the main corridor from phloem to aril and seed. Therefore, abortive seed cultivars in litchi are associated with the lower CWIN activities in the funicle and seed coat, due to the weak unloading capacity. Mutants of maize and rice with reduced CWIN activities produce smaller seeds, whereas elevation of CWIN activity in tomatoes increases the seed size [53,54,55]. Thus, CWIN play important roles in seed development. Increasing evidence indicate that hexes, especially glucose released from CWIN activity, not only provide nutrients, but also act as signals to activate the expression of seed development related genes [32].

Among the five *LcCWIN* genes, *LcCWIN5* is specifically expressed in anthers and pistils, while *LcCWIN2* is predominantly expressed in the funicle and the seed coat [49]. Silencing of *LcCWIN5* or *LcCWIN2* at early seed development result in impaired liquid endosperm development, smaller seeds, and/or higher seed abortion rate [49]. Therefore, it is suggested that the impaired endosperm development in abortive seed cultivars is due to decreased *CWIN* expression. In maize, the *Miniature1* (*ZmMn1*) encodes an endosperm specific CWIN, and its mutant impairs endosperm development by reducing the cell number [53]. Similarly, a larger endosperm correlates with expression changes of a CWIN gene, *OsGIF1* [54]. During early seed development, high glucose-to-sucrose ratios generally correlate with mitotic activity and favor cell division. In fava beans (*Vicia faba*), high CWIN activities correlate with high glucose levels and more cells are produced in the embryo [56]. In cotton early seed development, *GhCWIN1* plays important roles in transfer cells differentiation, endosperm nuclear division, and embryonic provascular development [57].

Embryo and endosperm are symplasmically isolated from the maternal seed coat, thus requiring the help of SUT or SWEET. Arabidopsis *sweet11;12;15* triple mutant causes a “wrinkled” seed phenotype, including retarded embryo development, reduced seed weight, and reduced starch and lipid content, implicating SWEET-mediated sucrose efflux in the transfer of sugars from seed coat to embryo [58]. Maize *ZmSWEET4c* transfers CWIN-derived hexoses across the basal endosperm transfer layer (BETL) as a key step in seed filling. Mutants of both maize *ZmSWEET4c* and its rice ortholog *OsSWEET4* are defective in seed filling [59]. In litchi, *LcSWEET2a* and *LcSWEET3b* are mainly expressed in seeds. The expression of *LcSWEET2a* and *LcSWEET3b* are higher in the big-seeded cultivar HY than that in the seed-aborting cultivar NMC. Moreover, *LcSWEET2a* and *LcSWEET3b* are mainly expressed in the funicle in HY, where the sucrose is apoplasmic phloem unloaded [27]. This research suggests that *SWEET* encodes hexose transporters acting downstream of CWIN that hydrolyzes phloem-derived sucrose. LcSWEET2a and LcSWEET3b appear to be responsible for transferring hexose from the funicle to endosperm for its development. Interestingly, the expression of *ZmMn1* and *ZmSWEET4c* is induced by glucose in the BETL, intimating that a ‘feed-forward’ mechanism, in which increasing glucose levels trigger enhanced membrane surface area, as well as increased capacity to hydrolyze sucrose and import hexoses into the BETL [59]. Moreover, *ZmSWEET4c* and *OsSWEET4* shows signs indicative of selection during domestication. SWEET4 is likely recruited during domestication to enhance sugar import into the endosperm in both maize and rice [59]. Further work is required to determine whether LcSWEET is also a target of selection during litchi domestication.

**Figure 3:**
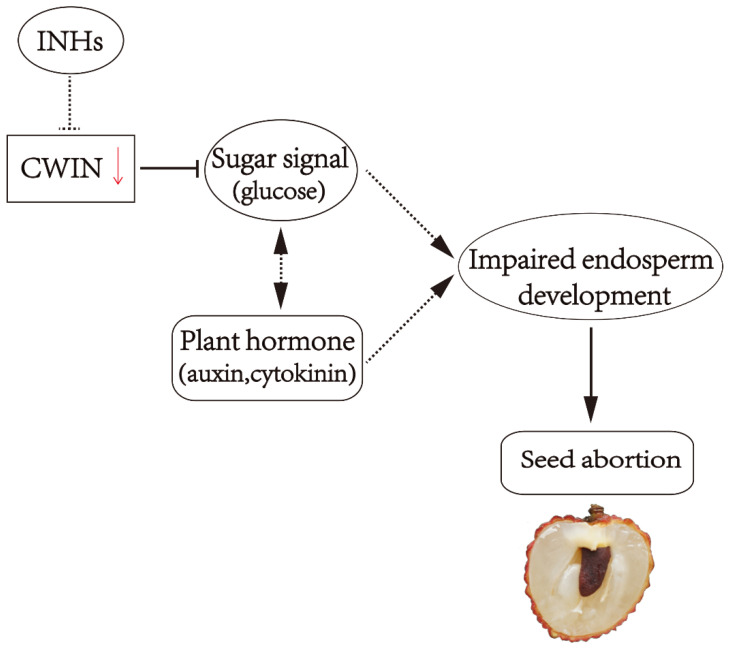
A hypothetic model for CWIN-mediated sugar availability and/or signal in the regulation of seed abortion in NMC.

In summary, we hypothesize that impaired liquid endosperm development is associated with the lower CWIN activity under certain intrinsic and/or external stimuli, resulting in seed abortion in NMC. In addition to impaired expression of *LcCWIN* gene, INHs that post-translationally regulate CWIN activity may be responsible for the decreased CWIN activity in NMC. Moreover, the CWIN-mediated sugar signal might interact with hormonal signaling pathways to regulate seed development in litchi (Figure 3).

## 5. Conclusions and Future Perspective

In this review, we focussed on the sugar transport, metabolism, and their involvement in litchi fruit development. As an important fruit quality parameter, sugar content and composition has also gained extensive attention for their critical roles in fruit development. We underlined the complexity of sugar transport and sugar accumulation during litchi aril development. Sugar content and composition vary considerably among cultivars. However, the molecular mechanism of the accumulation of high concentrations of glucose and fructose in the aril are still poorly characterized. Furthermore, factors that are involved in sugar transport, as well as the sugar metabolic enzymes, and the sink-source relationship, also need to be investigated to improve fruit quality and yield. Although high levels of quebrachitol have been detected in litchi, its physiological roles in the regulation of fruit development and adaptation to stress have not been well defined.

Beyond obtaining energy, the sugar status directly or indirectly affects fruit development through sugar signaling pathway. Here we reviewed the role of sugar metabolism in controlling important events in fruit abscission and seed development. CWIN activities are essential for seed development. However, it remains largely unknown as to how this is achieved. To answer this question, it will be essential to identify the molecule and biochemical pathways that are responsive to up- or downregulation of CWIN. Equally relevant is identifying the INHs that regulate CWIN activity at post-translational level. Interactions between the sugar and hormonal signaling pathway play a key role in fruit development. Further work is required to understand the regulatory mechanisms underlying sugar and hormone connection in controlling fruit growth, ripening, and abscission, as this will permit us to have a better control on fruit yield under abiotic stress. Answers to these questions will depend on the development of functional genomic tools in litchi that is recalcitrant to genetic transformation as well as long growth period.

## References

[1] White A.C., Rogers A., Rees M., Osborne C.P. (2016). How can we make plants grow faster? A source-sink perspective on growth rate. J. Exp. Bot..

[2] Yu S.M., Lo S.F., Ho T.D. (2015). Source-sink communication: Regulated by hormone, nutrient, and stress cross-signaling. Trends Plant Sci..

[3] Li Y.M., Forney C., Bondada B., Leng F., Xie Z.S. (2021). The molecular regulation of carbon sink strength in grapevine (*Vitis vinifera* L.). Front. Plant Sci..

[4] Kanayama Y. (2017). Sugar metabolism and fruit development in the tomato. Horticult. J..

[5] Durán-Soria S., Pott D.M., Osorio S., Vallarino J.G. (2020). Sugar signaling during fruit ripening. Front. Plant Sci..

[6] Hussain S.Z., Naseer B., Qadri T., Fatima T., Bhat T.A. (2021). Litchi (*Litchi chinensis*): Morphology, taxonomy, composition and health benefits. Fruits Grown in Highland Regions of the Himalayas.

[7] Hu B., Lai B., Wang D., Li J., Chen L., Qin Y., Wang H., Qin Y., Hu G., Zhao J. (2019). Three LcABFs are involved in the regulation of chlorophyll degradation and anthocyanin biosynthesis during fruit ripening in *Litchi chinensis*. Plant Cell Physiol..

[8] Wang H.C., Huang H.B., Huang X.M., Hu Z.Q. (2006). Sugar and acid compositions in the arils of *Litchi chinensis* Sonn.: Cultivar differences and evidence for the absence of succinic acid. J. Hortic. Sci. Biotechnol..

[9] Wang D., Zhao J., Hu B., Li J., Qin Y., Chen L., Qin Y., Hu G. (2018). Identification and expression profile analysis of the sucrose phosphate synthase gene family in *Litchi chinensis* sonn. PeerJ.

[10] Yang Z., Wang T., Wang H., Huang X., Qin Y., Hu G. (2013). Patterns of enzyme activities and gene expressions in sucrose metabolism in relation to sugar accumulation and composition in the aril of *Litchi chinensis* Sonn. J. Plant Physiol..

[11] Wu Z.C., Yang Z.Y., Li J.G., Chen H.B., Huang X.M., Wang H.C. (2016). Methyl-inositol, γ -aminobutyric acid and other health benefit compounds in the aril of litchi. Int. J. Food Sci. Nutr..

[12] Stern R.A., Gazit S., Janick J. (2003). The reproductive biology of the lychee. Horticultural Reviews.

[13] Li J.G., Huang H., Huang X. (2003). A revised division of the developmental stages in litchi fruit. Yuan Yi Xue Bao.

[14] Huang H., Xu J. (1983). The developmental patterns of fruit tissue and their correlative relationships in *Litchi chinensis* Sonn. Sci. Hortic..

[15] Cronje R.B. (2008). Effect of fruit development, maturity and harvesting of litchi (*Litchi chinensis* Sonn.) on postharvest fruit quality. Stewart Postharvest Rev..

[16] Wang H.C., Lai B., Huang X.M., Kumar M., Kumar V., Prasad R., Varma A. (2017). Litchi fruit set, development, and maturation. The Lychee Biotechnology.

[17] Huang H. (2001). Towards a better insight into the development of the arillate fruit of litchi, and longan. Acta Hort..

[18] Zhao L., Wang K., Wang K., Zhu J., Hu Z. (2020). Nutrient components, health benefits, and safety of litchi (*Litchi chinensis* Sonn.): A review. Compr. Rev. Food Sci. Food Saf..

[19] Wang H.C., Wu Z.C., Huang X.M., Hu G.B., Chen H.B. (2013). Determination of quebrachitol in *Litchi chinensis* and *Dimocarpus longan* in Sapindacea family. J. South China Agr. Univ..

[20] Wu Z.C., Zhang J.Q., Zhao J.T., Li J.G., Huang X.M., Wang H.C. (2018). The biosynthesis of quebrachitol, a transportable photosynthate, in *Litchi chinensis*. J. Exp. Bot..

[21] Jiang X., Lin H., Shi J., Neethirajan S., Lin Y., Chen Y., Wang H., Lin Y. (2018). Effects of a novel chitosan formulation treatment on quality attributes and storage behavior of harvested litchi fruit. Food Chem..

[22] Wang D., Zhao J., Qin Y., Qin Y., Hu G. Molecular cloning, characterization and expression profile of the sucrose synthase gene family in *Litchi chinensis*. Hortic. Plant J..

[23] Ma S., Li Y., Li X., Sui X., Zhang Z. (2019). Phloem unloading strategies and mechanisms in crop fruits. J. Plant Growth Regul..

[24] Falchi R., Bonghi C., Drincovich M.F., Famiani F., Lara M.V., Walker R.P., Vizzotto G. (2020). Sugar metabolism in stone fruit: Source-sink relationships and environmental and agronomical effects. Front. Plant Sci..

[25] Wang T.D., Zhang H.F., Wu Z.C., Li J.G., Huang X.M., Wang H.C. (2015). Sugar uptake in the aril of litchi fruit depends on the apoplasmic post-phloem transport and the activity of proton pumps and the putative transporter LcSUT4. Plant Cell Physiol..

[26] Chen L.Q., Cheung L.S., Feng L., Tanner W., Frommer W.B. (2015). Transport of sugars. Annu. Rev. Biochem..

[27] Xie H., Wang D., Qin Y., Ma A., Fu J., Qin Y., Hu G., Zhao J. (2019). Genome-wide identification and expression analysis of *SWEET* gene family in *Litchi chinensis* reveal the involvement of *LcSWEET2a/3b* in early seed development. BMC Plant Biol..

[28] Zhang Z., Zou L., Ren C., Ren F., Wang Y., Fan P., Li S., Liang Z. (2019). *VvSWEET10* mediates sugar accumulation in grapes. Genes.

[29] Stein O., Granot D. (2019). An overview of sucrose synthases in plants. Front. Plant Sci..

[30] Anur R.M., Mufithah N., Sawitri W.D., Sakakibara H., Sugiharto B. (2020). Overexpression of sucrose phosphate synthase enhanced sucrose content and biomass production in transgenic sugarcane. Plants.

[31] Cirilli M., Bassi D., Ciacciulli A. (2016). Sugars in peach fruit: A breeding perspective. Hortic. Res..

[32] Ruan Y.L. (2014). Sucrose metabolism: Gateway to diverse carbon use and sugar signaling. Annu. Rev. Plant Biol..

[33] Ruan Y.L., Jin Y., Yang Y.J., Li G.J., Boyer J.S. (2010). Sugar input, metabolism, and signaling mediated by invertase: Roles in development, yield potential, and response to drought and heat. Mol. Plant..

[34] Shen S., Ma S., Liu Y., Liao S., Li J., Wu L., Kartika D., Mock H.P., Ruan Y.L. (2019). Cell wall invertase and sugar transporters are differentially activated in tomato styles and ovaries during pollination and fertilization. Front. Plant Sci..

[35] Ru L., He Y., Zhu Z., Patrick J.W., Ruan Y.L. (2020). Integrating sugar metabolism with transport: Elevation of endogenous cell wall invertase activity up-regulates *SlHT2* and *SlSWEET12c* expression for early fruit development in tomato. Front. Genet..

[36] Ma M., Wang L., Zhang S., Guo L., Zhang Z., Li J., Sun L., Zhang S. (2020). Acid vacuolar invertase 1 (PbrAc-Inv1) and invertase inhibitor 5 (PbrII5) were involved in sucrose hydrolysis during postharvest pear storage. Food Chem..

[37] Wan H., Wu L., Yang Y., Zhou G., Ruan Y.L. (2018). Evolution of sucrose metabolism: The dichotomy of invertases and beyond. Trends Plant Sci..

[38] Klann E.M., Hall B., Bennett A.B. (1996). Antisense acid invertase (TIV1) gene alters soluble sugar composition and size in transgenic tomato fruit. Plant Physiol..

[39] Sakr S., Wang M., Dédaldéchamp F., Perez-Garcia M.D., Ogé L., Hamama L., Atanassova R. (2018). The sugar-signaling hub: Overview of regulators and interaction with the hormonal and metabolic network. Int. J. Mol. Sci..

[40] Li L., Sheen J. (2016). Dynamic and diverse sugar signaling. Curr. Opin. Plant Biol..

[41] Meitzel T., Radchuk R., McAdam E.L., Thormählen I., Feil R., Munz E., Hilo A., Geigenberger P., Ross J.J., Lunn J.E. (2021). Trehalose 6-phosphate promotes seed filling by activating auxin biosynthesis. New Phytol..

[42] Li C., Wang Y., Huang X., Li J., Wang H., Li J. (2015). An improved fruit transcriptome and the identification of the candidate genes involved in fruit abscission induced by carbohydrate stress in litchi. Front. Plant Sci..

[43] Zhao M., Li J. (2020). Molecular events involved in fruitlet abscission in litchi. Plants.

[44] Li C., Wang Y., Huang X., Li J., Wang H., Li J. (2013). *De novo* assembly and characterization of fruit transcriptome in *Litchi chinensis*. Sonn and analysis of differentially regulated genes in fruit in response to shading. BMC Genom..

[45] Kuang J.F., Wu J.Y., Zhong H.Y., Li C.Q., Chen J.Y., Lu W.J., Li J.G. (2012). Carbohydrate stress affecting fruitlet abscission and expression of genes related to auxin signal transduction pathway in litchi. Int. J. Mol. Sci..

[46] Denisov Y., Glick S., Zviran T., Ish-Shalom M., Levin A., Faigenboim A., Cohen Y., Irihimovitch V. (2017). Distinct organ-specific and temporal expression profiles of auxin-related genes during mango fruitlet drop. Plant Physiol. Biochem..

[47] Xie D.R., Ma X.S., Rahman M.Z., Yang M.C., Huang X.M., Li J.G., Wang H.C. (2019). Thermo-sensitive sterility and self-sterility underlie the partial seed abortion phenotype of *Litchi chinensis*. Sci. Hortic..

[48] Orozco-Arroyo G., Paolo D., Ezquer I., Colombo L. (2015). Networks controlling seed size in Arabidopsis. Plant Reprod..

[49] Zhang J., Wu Z., Hu F., Liu L., Huang X., Zhao J., Wang H. (2018). Aberrant seed development in *Litchi chinensis* is associated with the impaired expression of cell wall invertase genes. Hortic. Res..

[50] Lafon-Placette C., Köhler C. (2014). Embryo and endosperm, partners in seed development. Curr. Opin. Plant Biol..

[51] Sekhon R.S., Hirsch C.N., Childs K.L., Breitzman M.W., Kell P., Duvick S., Spalding E.P., Buell C.R., de Leon N., Kaeppler S.M. (2014). Phenotypic and transcriptional analysis of divergently selected maize populations reveals the role of developmental timing in seed size determination. Plant Physiol..

[52] Yang Z.Y., Zhang J.Q., Wang T.D., Huang X.M., Hu G.B., Wang H.C. (2014). Does acid invertase regulate the seed development of *Litchi Chinensis*?. Acta Hortic..

[53] Cheng W.H., Taliercio E.W., Chourey P.S. (1996). The *Miniature1* seed locus of maize encodes a cell wall invertase required for normal development of endosperm and maternal cells in the pedicel. Plant Cell.

[54] Wang E.T., Wang J., Zhu X., Hao W., Wang L., Li Q., Zhang L., He W., Lu B., Lin H. (2008). Control of rice grain-filling and yield by a gene with a potential signature of domestication. Nat. Genet..

[55] Jin Y., Ni D.A., Ruan Y.L. (2009). Posttranslational elevation of cell wall invertase activity by silencing its inhibitor in tomato delays leaf senescence and increases seed weight and fruit hexose level. Plant Cell.

[56] Weschke W., Panitz R., Gubatz S., Wang Q., Radchuk R., Weber H., Wobus U. (2003). The role of invertases and hexose transporters in controlling sugar ratios in maternal and filial tissues of barley caryopses during early development. Plant J..

[57] Wang L., Ruan Y.L. (2012). New insights into roles of cell wall invertase in early seed development revealed by comprehensive spatial and temporal expression patterns of *GhCWIN1* in cotton. Plant Physiol..

[58] Chen L.Q., Lin I.W., Qu X.Q., Sosso D., McFarlane H.E., Londoño A., Samuels A.L., Frommer W.B. (2015). A cascade of sequentially expressed sucrose transporters in the seed coat and endosperm provides nutrition for the Arabidopsis embryo. Plant Cell.

[59] Sosso D., Luo D., Li Q.B., Sasse J., Yang J., Gendrot G., Suzuki M., Koch K.E., McCarty D.R., Chourey P.S. (2015). Seed filling in domesticated maize and rice depends on SWEET-mediated hexose transport. Nat. Genet..

